# A new adjustable parallel drill guide for internal fixation of femoral neck fracture: a developmental and experimental study

**DOI:** 10.1186/s12891-015-0845-2

**Published:** 2016-01-11

**Authors:** Varah Yuenyongviwat, Pakjai Tuntarattanapong, Boonsin Tangtrakulwanich

**Affiliations:** Department of Orthopaedic Surgery and Physical Medicine, Faculty of Medicine, Prince of Songkla University, Songkhla, 90110 Thailand

**Keywords:** Femoral neck fracture, Drill guide, Internal fixation

## Abstract

**Background:**

Internal fixation is one treatment for femoral neck fracture. Some devices and techniques reported improved accuracy and decreased fluoroscopic time. However, these are not widely used nowadays due to the lack of available special instruments and techniques. To improve the surgical procedure, the authors designed a new adjustable drill guide and tested the efficacy of the device.

**Methods:**

The authors developed a new adjustable drill guide for cannulated screw guide wire insertion for multiple screw fixation. Eight orthopaedic surgeons performed the experimental study to evaluate the efficacy of this device. Each surgeon performed guide wire insertion for multiple screw fixation in six synthetic femurs: three times with the new device and three times with the conventional technique. The fluoroscopic time, operative time and surgeon satisfaction were evaluated.

**Results:**

In the operations with the new adjustable drill guide, the fluoroscopic and operative times were significantly lower than the operations with the conventional technique (*p* < 0.05). The mean score for the level of satisfaction of this device was also statistically significantly better (*p* = 0.02) than the conventional technique.

**Conclusions:**

The fluoroscopic and operative times with the new adjustable drill guide were reduced for multiple screw fixation of femoral neck fracture and the satisfaction of the surgeons was good.

## Background

Internal fixation is the standard treatment of femoral neck fracture in young patients and non-displaced or impacted femoral neck fracture in the elderly [1, 2]. The key success factor of this fixation depends on the quality of the bone, number of screws, and screw position in the femoral neck [3, 4]. Parallel three-screw fixation is better than two-screw fixation because three-screw fixation has greater resistance to load and less fracture displacement [5]. The other factor is the position of the screws. A greater separation between the screws results in better fixation stability [6]. In addition, each screw must be inserted parallel to each other and form an inverted equilateral triangle configuration [7]. The need for very precise screw placement requires a meticulous surgical technique that usually requires a prolonged operative time and the surgeon has to deal with high radiation exposure.

Recently, several devices and techniques were developed to improve the accuracy and minimize fluoroscopic time. Computer-based navigation techniques [8–10] using a cannulated screw as the drill guide [11] were reported to reduce radiation time and improve accuracy. However, these techniques are not widely used because of the limitation on the higher costs of the special instruments.

Our group designed a new adjustable drill guide device. The aim of this study was to compare the fluoroscopic time, operative time, and surgeon satisfaction of the new device with the conventional technique in cannulated guide wire insertion for multiple screw fixation of femoral neck fractures.

## Methods

### Design and development

**Fig. 1:**
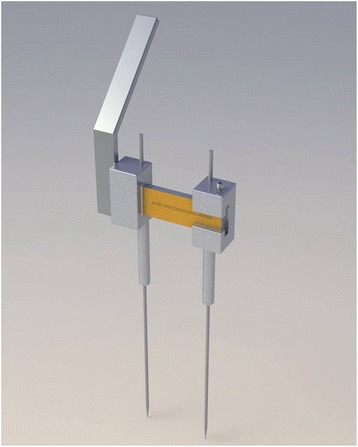
Three-dimensional model of the new adjustable drill guide prototype

An orthopaedic surgeon and a design engineer developed the new adjustable drill guide design. After the concept and information were analysed, a three-dimensional model was created (Fig. 1).

The concept for development of this drill guide was based on the drill guide helping the surgeon insert the cannulated guide wire with an easily adjustable separation distance from the reference guide wire and produce good stability during insertion of the cannulated guide wire.

**Fig. 2:**
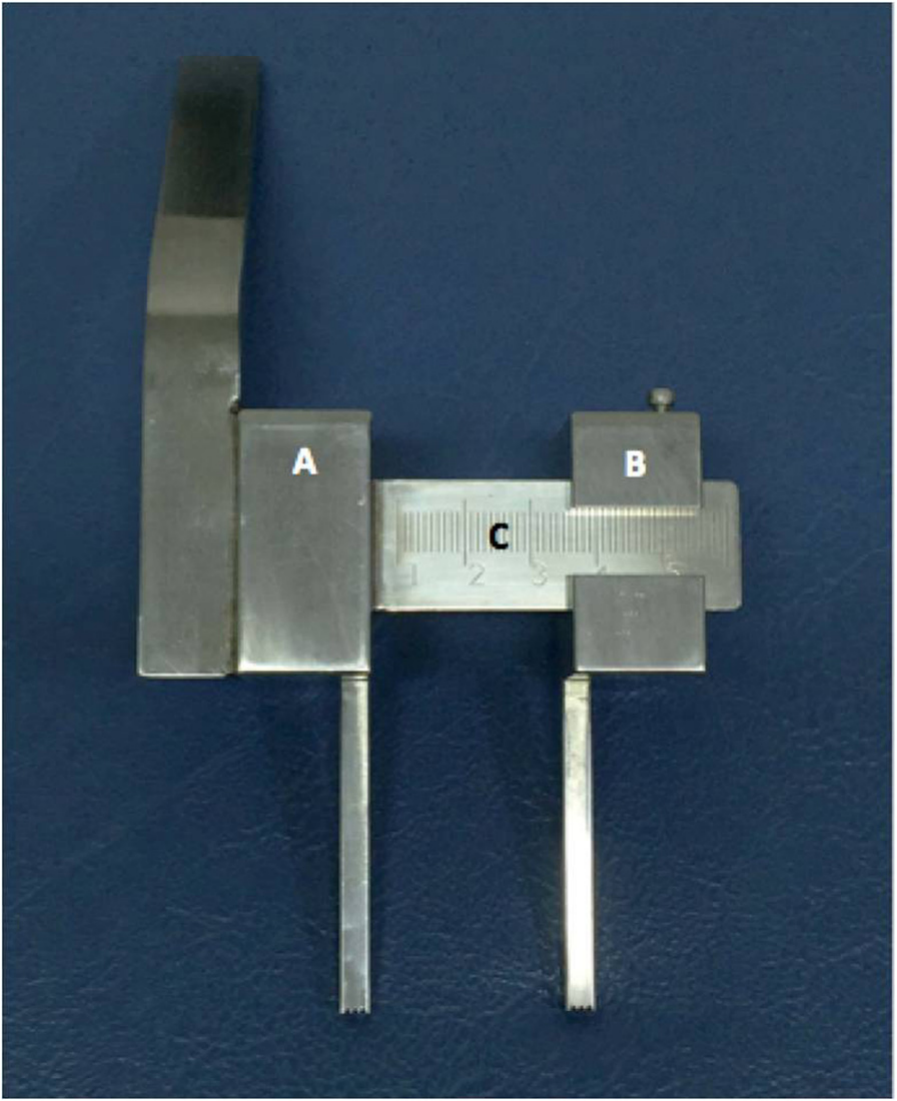
New adjustable drill guide consists of the reference part (**a**) and drilling part (**b**) which slides along the extension rod (**c**)

**Fig. 3:**
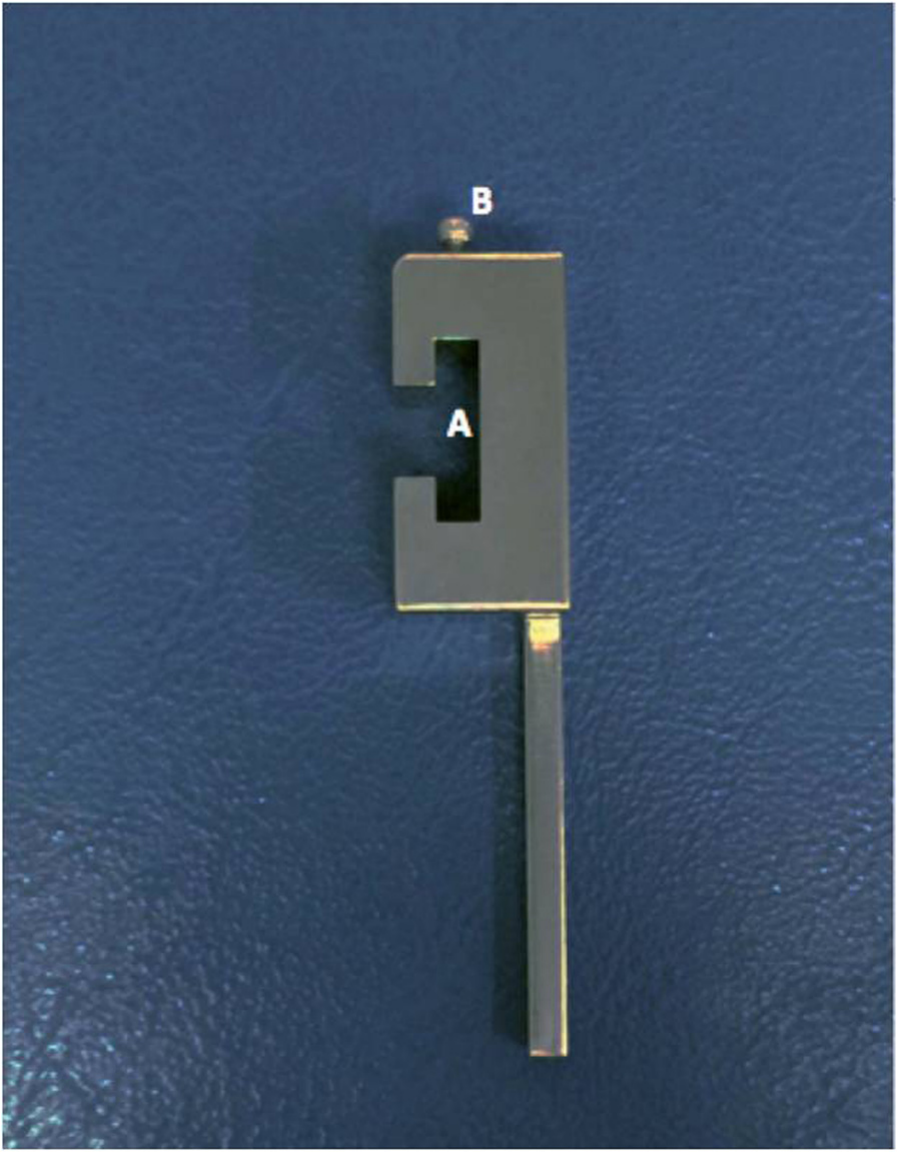
Side view of the drilling part illustrates the mid-body slot (**a**) and locking knob (**b**)

The drill guide consists of two parts: the reference part and the drilling part (Fig. 2). The reference part has a handle, reference tube for cannulated guide wire insertion and a square extension rod to support the drilling part that can slide along the extension rod (Fig. 3). There are scale numbers on the extension rod for length adjustment. The drilling part is slotted in the middle of the body to allow it to slide on the extension rod. The drilling part is able to move along the extension rod to maintain the drill guide tube parallel to the reference cannulated guide wire. A locking knob can tighten the drilling part to fix the position after adjustment of the distance. At the end of both guide rods, there are spikes to grip the bone to prevent drill slippage while drilling.

### Simulated operation testing

This study was approved by the Ethics Committee and Institutional Review Board of the Faculty of Medicine at Prince of Songkla University, Songkhla, Thailand. This study was conducted in synthetic bones and not related to human subjects. So, an informed consent was unnecessary according to institutional regulations.

The experimental study evaluated the efficacy of the new drill guide compared to the conventional technique. The intraoperative setting was a simulated multiple screw fixation in femoral neck fracture with the patient in the supine position. The synthetic femoral bone made of foam cortical shell with cancellous inner material (Sawbones™, Pacific Research Laboratories, Inc., Vashon, Washington, USA) was placed on the operative table and fixed with a synthetic bone holder. The synthetic bone was covered with sponges to simulate the soft tissue compartment.

Eight orthopaedic surgeons were included in this study. They were given instructions and then practiced the technique to use this new drill guide. Each surgeon performed cannulated guide wire insertion for multiple screw fixation in six femurs using three guide wires for three-screw cannulated screw fixation in each femur. They performed the operation three times with the new drill guide and three times with the conventional technique. In the conventional technique group, guide wires were inserted using a standard parallel wire guide (Synthes 312.71 Parallel Wire Guide, Synthes, Paoli, Pennsylvania, USA) and protection sleeve. The operations were performed under C-arm fluoroscopy (Philips BV Libra, Philips Medical Systems North America Co., Bothell, Washington, USA) in the same operative theater environment.

**Fig. 4:**
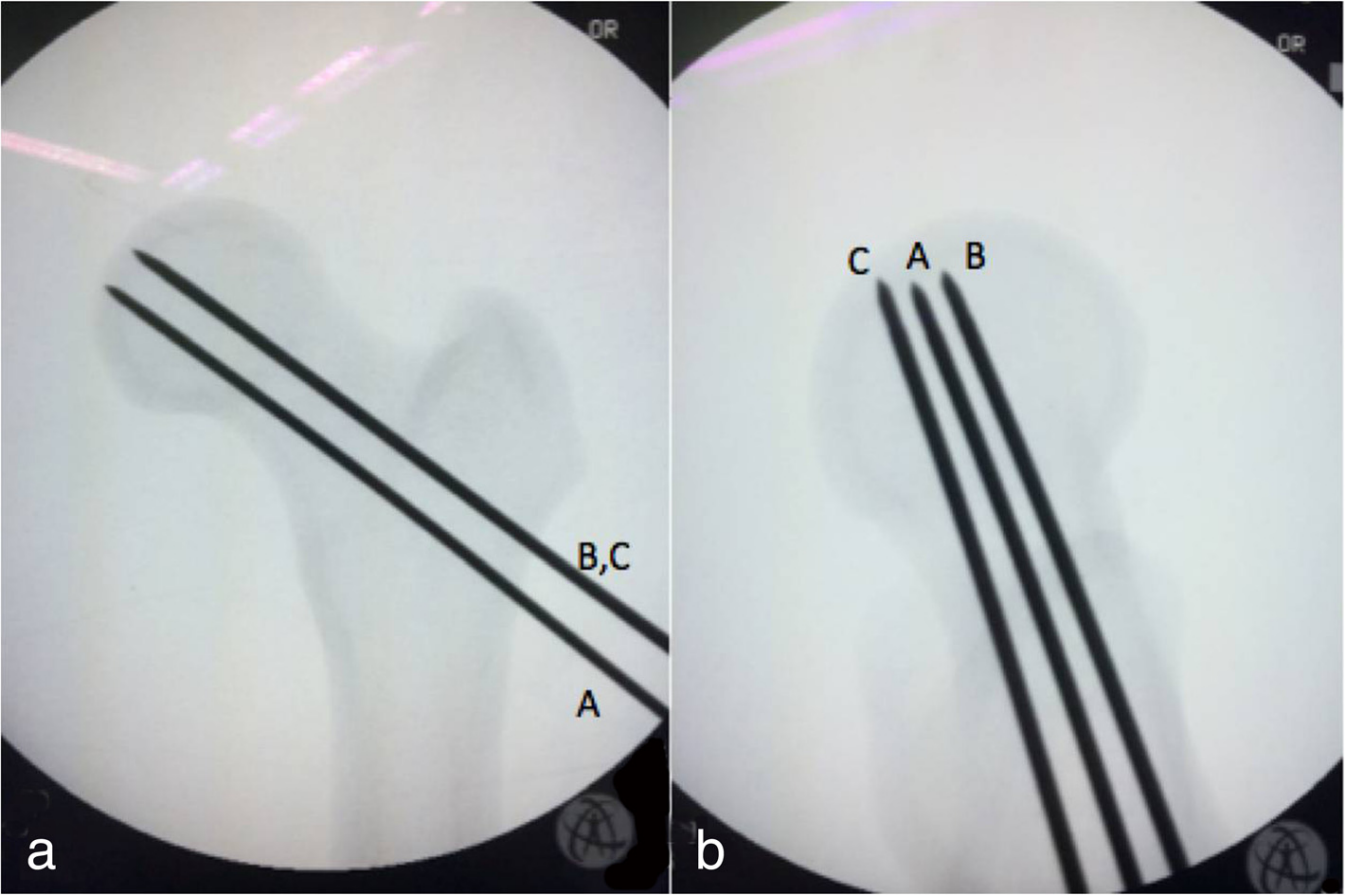
Guide wire position in anteroposterior (**a**) and lateral (**b**) fluoroscopic views: first guide wire (*A*), second guide wire (*B*), third guide wire (*C*)

The goal of each operation was to insert successfully three 2.0-mm cannulated guide wires to the target position. Three guide wires were inserted in the inverted triangle configuration. The first guide wire position was superior to the inner cortex of the inferior femoral neck (4 ± 1 mm) and parallel to the femoral neck in the anteroposterior view (Fig. 4-a). In the lateral fluoroscopic view, the first guide wire was in the middle of the femoral neck (±1 mm) and parallel to the femoral neck (Fig. 4-b). The second and third guide wire positions were in the middle of the femoral neck (±1 mm) and parallel to the femoral neck in the anteroposterior view. In the lateral view, the second guide wire was at 4 ± 1 mm posterior to the inner cortex of the anterior femoral neck and the third guide wire was at 4 ± 1 mm anterior to the inner cortex of the posterior femoral neck. The depths of all guide wire insertions were at the subchondral bone of the femoral head. The fluoroscopic time, total operative time, and surgeon satisfaction were recorded. The fluoroscopic time was recorded from the fluoroscopic machine and the level of satisfaction was evaluated by a visual analogue scale (VAS). The VAS consisted of a 10 cm line where 0 indicated no satisfaction at all and 10 represented total satisfaction.

### Statistical methods

The statistical analysis was performed using R software version 3.1.0 (R Foundation). In order to compare fluoroscopic time and operative time, the differences between the groups used the Wilcoxon signed-rank test. The student *t*-test was used for comparison of satisfaction scores. Statistical significance was considered when *P* was less than 0.05.

A statistical power analysis was performed for sample size estimation based on data from a previous study [10]. To detect a 10 % difference in fluoroscopic time with a power set to 0.9 and a significance level set to 0.05, nine samples per group were required.

## Results

The fluoroscopic and operative times are presented in Table 1. The fluoroscopic time with the new drill guide was significantly lower than the operation with the conventional technique (*p* = 0.007). The operative time was also significantly lower in the operation with the new drill guide (*p* = 0.01). The mean level of satisfaction of the operation with the new drill guide was 9 (SD 0.93) which was statistically significantly higher (*p* = 0.02) than the satisfaction score of the conventional technique (7.18, SD 0.92).

**Table 1:** Fluoroscopic and operative times of the operations

	Conventional (24)	New device (24)	*p* value
Fluoroscopic time (seconds)	28.5 (13–45)	22 (10–50)	0.007
Operative time (minutes)	16 (9–28)	12 (7–24)	0.01

## Discussion

Femoral neck fracture is a fracture that produces significant morbidity and mortality. The majority of fractures occur in the elderly from low-energy trauma and a low incidence of fractures occurs in younger patients from high-energy trauma [12]. There are two main options for treatment of femoral neck fracture: internal fixation and arthroplasty [13].

Multiple screw fixation is the choice of treatment for non-displacement, impacted fracture in the elderly and also displaced fracture in young patients [14]. Screw position and configuration are the crucial parts of a successful outcome. Selvan reported a biomechanical study of configuration of multiple screws for the fixation of intracapsular hip fractures. The study showed that triangular configurations of three parallel screws could resist a higher load than other screw configurations [7]. Due to the high precision needed for screw placement, this operation usually requires a long operative time and high radiation exposure to the surgeon.

There were reports of new surgical techniques and devices to improve the operating procedure and decrease the radiation exposure and operative times. Liebergall et al. reported a comparison study between a computer-based navigation technique and the conventional fluoroscopy technique. The results showed that the navigation group had better screw positions and had a tendency for a fewer number of reoperations and overall complications [8]. Kendoff et al. also reported results of an integration of the parallel drill guide and navigation module compared with the conventional technique. The study found a reduction of radiation time in the navigation group but the operative time increased [9]. Another report showed a significant reduction in operative time by using a cannulated screw as a drill guide and sleeve compared with the conventional technique [11].

This present study demonstrated the advantages of the new adjustable drill guide in comparison with the conventional technique. Since the distance for drilling is easily adjustable with the new stable drill guide, high accuracy for parallel drilling can be maintained. The spikes at the tip of the sleeve also prevent drill guide slippage as the drill starts through the femoral cortex. The new drill guide has a low learning curve to become familiar with the device and most surgeons in this study were satisfied with this new device. This new adjustable drill guide might also be applied to other operations which require parallel drilling from a reference point such as odontoid screw fixation or anti-rotational screw for dynamic hip screw fixation [11, 15].

## Conclusions

In this experimental study, the new adjustable drill guide reduced fluoroscopic and operative times in multiple screw fixation for femoral neck fracture with good surgeon satisfaction. However, further clinical studies should be conducted to evaluate the efficacy of this new device in clinical practice.

## References

[1] Asnis SE, Wanek-Sgaglione L (1994). Intracapsular fractures of the femoral neck. Results of cannulated screw fixation. J Bone Joint Surg Am.

[2] Ravikumar KJ, Marsh G (2000). Internal fixation versus hemiarthroplasty versus total hip arthroplasty for displaced subcapital fractures of femur—13 year results of a prospective randomised study. Injury.

[3] Tan V, Wong KL, Born CT, Harten R, DeLong WG (2007). Two-screw femoral neck fracture fixation: a biomechanical analysis of 2 different configurations. Am J Orthop.

[4] Gurusamy K, Parker MJ, Rowlands TK (2005). The complications of displaced intracapsular fractures of the hip: the effect of screw positioning and angulation on fracture healing. J Bone Joint Surg (Br).

[5] Maurer SG, Wright KE, Kummer FJ, Zuckerman JD, Koval KJ (2003). Two or three screws for fixation of femoral neck fractures?. Am J Orthop.

[6] Kloen P, Rubel IF, Lyden JP, Helfet DL (2003). Subtrochanteric fracture after cannulated screw fixation of femoral neck fractures: a report of four cases. J Orthop Trauma.

[7] Selvan VT, Oakley MJ, Rangan A, Al-Lami MK (2004). Optimum configuration of cannulated hip screws for the fixation of intracapsular hip fractures: a biomechanical study. Injury.

[8] Liebergall M, Ben-David D, Weil Y, Peyser A, Mosheiff R (2006). Computerized navigation for the internal fixation of femoral neck fractures. J Bone Joint Surg Am.

[9] Kendoff D, Hüfner T, Citak M, Geerling J, Maier C, Wesemeier F (2006). Implementation of a new navigated parallel drill guide for femoral neck fractures. Comput Aided Surg.

[10] Kendoff D, Hüfner T, Citak M, Maier C, Wesemeier F, Pearle A (2006). A new parallel drill guide for navigating femoral neck screw placement. Development and evaluation. Unfallchirurg.

[11] Tai T-W, Lien F-C, Lee P-Y, Jou I-M, Lin C-J, Huang Y-H. Using a cannulated screw as a drill guide and sleeve: a simple technique for multiple-screw fixation for intracapsular femoral neck fracture. Orthopedics. 2010;33. 10.3928/01477447-20100625-05 20704114

[12] Johnell O, Kanis JA (2004). An estimate of the worldwide prevalence, mortality and disability associated with hip fracture. Osteoporos Int.

[13] Florschutz AV, Langford JR, Haidukewych GJ, Koval KJ (2015). Femoral neck fractures. J Orthop Trauma.

[14] Bout CA, Cannegieter DM, Juttmann JW (1997). Percutaneous cannulated screw fixation of femoral neck fractures: The three point principle. Injury.

[15] Wu A-M, Wang X-Y, Xia D-D, Luo P, Xu H-Z, Chi Y-L. A novel technique of two-hole guide tube for percutaneous anterior odontoid screw fixation. Spine J. 2015;11. 10.1016/j.spinee.2015.02.013 25681228

